# A Role for the Immediate Early Gene Product c-fos in Imprinting T Cells with Short-Term Memory for Signal Summation

**DOI:** 10.1371/journal.pone.0018916

**Published:** 2011-04-28

**Authors:** Carolyn E. Clark, Milena Hasan, Philippe Bousso

**Affiliations:** 1 Institut Pasteur, Dynamics of Immune Responses Unit, Paris, France; 2 Inserm U668, Paris, France; 3 Institut Pasteur, Center For Human Immunology, Paris, France; University Paris Sud, France

## Abstract

T cells often make sequential contacts with multiple DCs in the lymph nodes and are likely to be equipped with mechanisms that allow them to sum up the successive signals received. We found that a period of stimulation as short as two hours could imprint on a T cell a “biochemical memory” of that activation signal that persisted for several hours. This was evidenced by more rapid induction of activation markers and earlier commitment to proliferation upon subsequent stimulation, even when that secondary stimulation occurred hours later. Upregulation of the immediate early gene product c-fos, a component of the AP-1 transcription factor, was maximal by 1–2 hours of stimulation, and protein levels remained elevated for several hours after stimulus withdrawal. Moreover, phosphorylated forms of c-fos that are stable and transcriptionally active persisted for a least a day. Upon brief antigenic stimulation in vivo, we also observed a rapid upregulation of c-fos that could be boosted by subsequent stimulation. Accumulation of phosphorylated c-fos may therefore serve as a biochemical fingerprint of previous suboptimal stimulation, leaving the T cell poised to rapidly resume its activation program upon its next encounter with an antigen-bearing DC.

## Introduction

Naïve T cells continuously exit the circulation to crawl though lymph nodes (LNs), scanning antigen-presenting cells (APCs), primarily dendritic cells (DCs), for peptides presented in the groove of MHC molecules (pMHC complexes). Several hours of TCR stimulation are required for efficient T cell activation and commitment to proliferation [1], [2], [3]. It is not clear, however, whether the hours of long-term contacts between T cells and DCs need necessarily be continuous. Recent in vitro studies have suggested that T cells are equipped with mechanisms that allow them to integrate, in an additive manner, the signals received during periods of interrupted stimulation [4]. Using proliferation as a readout, one study demonstrated that naïve T cells can sum up signals delivered during two 7 hour-long periods of stimulation separated by a rest period, even one lasting nearly a day [5]. In a complementary study, the production of IFN-γ by T cell clones was found to be proportional to the duration of the TCR stimulation even when the intervals of stimulation were alternated with intervals of rest [6]. The ability of T cells to thus recall short, suboptimal stimulations has been termed “biochemical T cell memory” to distinguish it from classical T cell memory [7].

Whether this mode of activation also operates in vivo is an open question, but studies using multi-photon laser microscopy have provided clues that it might be the case [8]. These studies, involving real-time observation of interactions of T cells with DCs within explanted LNs or in the LNs of anesthetized mice, have revealed that T cells often have the opportunity to engage in successive and diverse interactions with Ag-bearing DCs. In some settings, early during antigen exposure the interactions may be predominantly transient, lasting several minutes, while later on during priming they progress to become stable contacts lasting several hours [9], [10]. Activated T cells can also re-interact with Ag-bearing DCs in the late phase of priming [11]. It remains to be firmly established whether signals received at these different stages are additive. In this respect, we have previously shown that T cells integrate signals received during late interactions with DCs to sustain CD25 expression and become IFN-γ producers [11]. Furthermore, T cells stimulated in vitro for 24 h respond more rapidly upon adoptive transfer than their naïve counterparts, suggesting that their initial activation program was ‘memorized’ [12]. However, it remains unclear whether T cells integrate short, suboptimal signals during serial contacts in vivo, in particular prior to commitment to proliferation.

Another important question relates to the molecular basis underlying such biochemical memory. A number of non-mutually exclusive mechanisms could potentially endow the T cell with this ability. While most proximal signaling intermediates are likely to be lost within seconds of cessation of TCR engagement, it has recently been shown that the feedback regulation of SOS (Son of Sevenless) results in hysteresis in Ras activation, a mechanism that conferred lymphocytes primed by a first stimulation with the ability to respond to weak stimulation even 5–10 min after removal of the initial stimulus [13]. Early epigenetic modifications are also likely to participate in such T cell biochemical memory [14]. Recent computational modeling has suggested that accumulation of transcription factors regulated by a positive feedback loop might help T cells memorize past activation signals [7]. One such transcription factor is AP-1, which plays an important role in the T cell activation program and cell cycle progression [15], [16], [17]. C-fos, a component of this dimeric transcription factor, is an immediate early gene (IEG) product rapidly induced upon T cell activation [18], [19]. Whether c-fos could act as a molecular counter of intermittent TCR stimulation is an intriguing possibility.

Here we provide new evidence that T cells have the ability to sum up interrupted suboptimal stimulations both in vitro and in vivo. In addition, we show that the total c-fos pool remains elevated in the nucleus of activated T cells for a few hours following signal withdrawal, while a key phosphorylated form remains stable for at least one day. Finally, we provide evidence that c-fos increases incrementally upon serial in vivo antigenic stimulations. We propose that phosphorylated c-fos may serve as a biochemical fingerprint of previous suboptimal stimulation, leaving the T cell poised to rapidly resume its activation program upon its next encounter with an antigen-bearing DC.

## Results and Discussion

### T cells integrate intermittent signals in vitro

**Figure 1:**
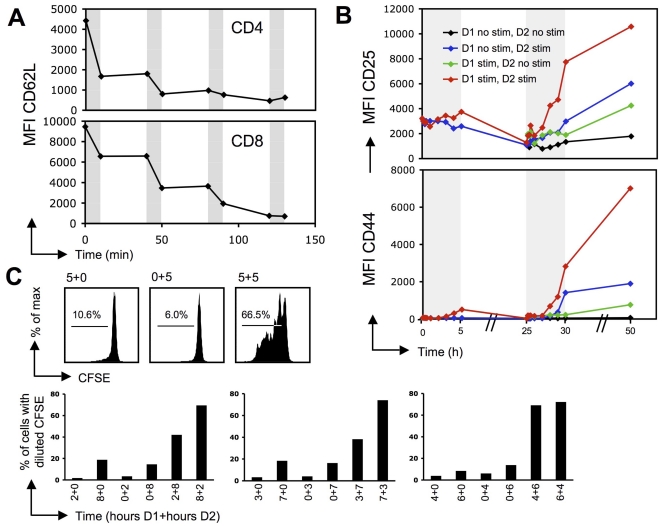
T cells have the ability to sum up periods of sub-optimal stimulation in vitro. A. Splenocytes were stimulated with anti-CD3/anti-CD28 magnetic beads for intervals of 10 minutes (indicated by grey shading) separated by rest periods of 30 minutes. Shedding of CD62L on CD4 T cells (top panel) and CD8 T cells (bottom panel) occurs exclusively during the periods of incubation with the beads, suggesting a rapid and complete cessation of TCR signaling upon removal of the beads. B. CD4 T cells, when stimulated (grey shading) with anti-CD3/anti-CD28 beads for 5 h on day 1 (D1), demonstrate a memory-like response in induction of the activation markers CD25 (top panel) and CD44 (bottom panel) upon secondary stimulation for 5 h on day 2 (D2). C. Top: OT-I splenic T cells do not divide when stimulated with anti-CD3/anti-CD28 beads for 5 h alone either on day 1 (left panel) or day 2 (middle panel), but commit to proliferation after an additive stimulus of 5 h on day 1 and 5 h on day 2 separated by a 20 h rest period (right panel). Numbers indicate percentages of cells with diluted CFSE. Bottom: combinations of two periods of stimulation adding up to 10 h show additive effects on proliferation. Stimulations of day 1 (D1) and day 2 (D2) were separated by a rest period of 20 h. Results are representative of at least 2 independent experiments.

To test the parameters for the programming of T cell biochemical memory, we first used anti-CD3/anti-CD28-conjugated magnetic beads to sequentially stimulate T cells in vitro since this system allowed us to easily control the initiation and arrest of each period of stimulation. To validate that the removal of beads effectively arrested signaling, we evaluated the shedding of CD62L as a rapid readout of TCR signaling that could be readily detected by flow cytometry. Indeed, CD62L shedding has been shown to occur within minutes of TCR signaling [20], [21] and has been used previously as a marker of recent TCR engagement in vivo [22], [23]. Shedding of CD62L was evident after only 10 minutes of stimulation in our system and occurred virtually exclusively during periods of stimulation and not during periods of rest, indicating that TCR signaling was rapidly aborted when the beads were removed with a magnet (Figure 1A). Having established a simple in vitro system, we then tested for evidence of biochemical memory using as readouts both expression of activation markers and proliferation. When T cells were stimulated for a period of 5 h, they modestly upregulated the activation markers CD25 and CD44, but by 20 h after removal of the beads, the expression of these markers had returned to baseline levels and was indistinguishable from that of control cells that had remained in culture without stimulation. Upon secondary stimulation, however, the prestimulated cells demonstrated a “memory-like” response in that they upregulated CD25 and CD44 again more rapidly and more robustly than they had done during the primary stimulation and as compared to their counterpart cells that were previously unstimulated (Figure 1B). These results indicate that the first stimulation imprinted certain changes on the T cells that remained stable throughout the rest period of 20 h.

We titrated the number of hours of stimulation necessary in this system to induce T cell proliferation by 72 h later and found it to be about 10 h (data not shown). However, the 10 h did not need to be continuous, but could be broken up into two shorter periods of stimulation, each of which would be insufficient on its own to cause commitment to proliferation. For example, one 5 h period of stimulation given either on day 1 or day 2 of culture did not promote proliferation in T cells by day 5 as measured by CFSE dilution, but if the first 5 h stimulation was followed by another 5 h stimulation the following day, this protocol induced robust proliferation (Figure 1C). These results resembled those of a previous report demonstrating T cell integration of two sub-optimal periods of in vitro stimulation on antibody-coated plates to induce proliferation [5]. To further extend these findings, we varied the duration of each stimulus to test the respective contributions of each and found that the initial stimulation seemed to contribute more to the activation program than the subsequent stimulation. As shown in Figure 1C, an initial stimulation of 2 h was sufficient to endow the T cells with a biochemical memory. However, the longer the first stimulation, the more efficient was the proliferation (Figure 1C), raising the possibility that optimal summation may be achieved after certain proteins, such as signaling intermediates, transcription factors and/or cytokines, accumulate beyond a certain threshold.

### T cells can integrate intermittent signals in vivo

**Figure 2:**
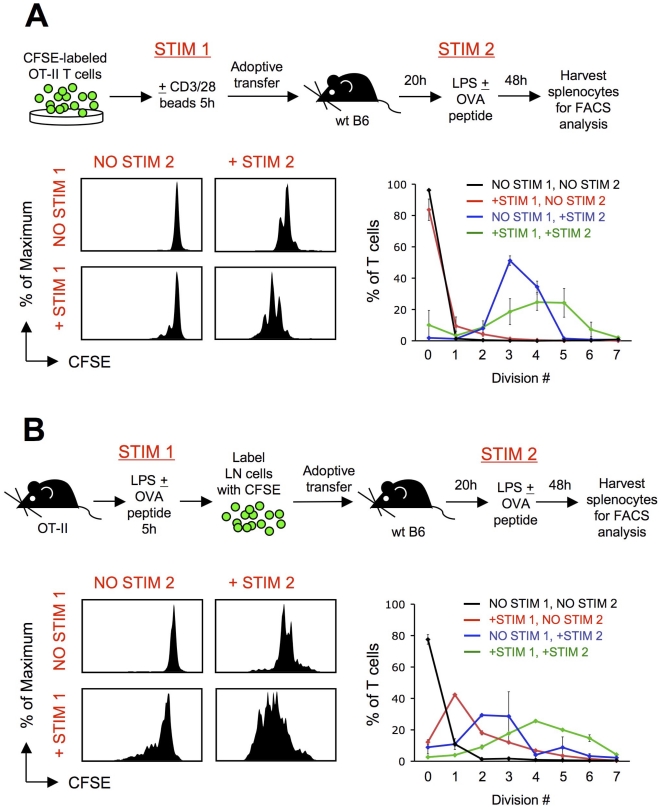
T cells sum up intermittent TCR stimulation in vivo. OT-II T cells stimulated 5 h in vitro or in vivo and rested in a naïve recipient display a proliferative advantage upon subsequent antigen encounter compared to their unstimulated counterparts. A. OT-II T cells were stimulated in vitro for 5 h with anti-CD3/anti-CD28 beads, or left unstimulated, then labeled with CFSE before transfer into wild-type C57BL/6 hosts. A secondary stimulation was provided 20 h later by injection of 50 µg LPS followed by OVA^323–339^ peptide (or PBS control). Recipient spleens were harvested 48 hours later to assess transferred cells for proliferation. OT-II T cells that had been prestimulated underwent additional rounds of division, evident by CFSE dilution, as compared to their non-prestimulated counterparts. Graph shows the percentage of recovered T cells that had undergone the indicated number of cell divisions. Results are mean±sem and are representative of 3 independent experiments. B. OT-II T cells were stimulated in vivo for 5 h by injecting LPS±OVA^323–339^ peptide into OT-II mice and harvesting LN cells 5 h later. Cells were labeled with CFSE before transfer into wild-type C57BL/6 hosts. A secondary stimulation was provided by injection with LPS±OVA^323–339^ peptide 20 h later. Recipient spleens were harvested 48 h later to assess transferred cells for proliferation. Graph shows the percentage of recovered T cells that had undergone the indicated number of cell divisions. Results are mean±sem and are representative of 2 independent experiments.

It remains to be established whether signal summation also occurs in vivo. We first set out to determine whether a short period of in vitro prestimulation would be “remembered” by T cells once they had been transferred in vivo, thus conferring on these cells a head start in proliferation. To this end, we injected mice with CFSE-labeled OT-II TCR transgenic T cells that had been prestimulated or not for 5 h with anti-CD3/anti-CD28-conjugated beads. The following day, the recipients were injected with OVA peptide, and OT-II T cell proliferation was analyzed two days later. As shown in Figure 2A, OT-II T cells that had been prestimulated prior to adoptive transfer had undergone approximately two additional rounds of division as compared to their non-prestimulated counterparts. Next, we assessed whether this proliferative advantage would also be conferred if the initial short stimulation occurred in vivo in response to antigen. To this end, OT-II T cells were prestimulated for 5 h in vivo by injecting OT-II mice with OVA peptide. T cells were harvested, labeled with CFSE and adoptively transferred into naive recipients. The next day, recipients were injected with OVA peptide, and proliferation was assessed 48 h later (Figure 2B). In accordance with the previous experiments using in vitro stimulation, the OT-II T cells that had been prestimulated for 5 h in vivo had undergone additional rounds of division, demonstrating that the first period of stimulation had been integrated into their activation program and had enabled them to start proliferating sooner or more efficiently (Figure 2B). Thus, while T cells' ability to sum up intermittent signals has been convincingly demonstrated in vitro, our results provide strong evidence that such a mode of signal integration also operates in vivo.

### A phosphorylated form of c-fos remains elevated at least one day after cessation of TCR engagement

**Figure 3:**
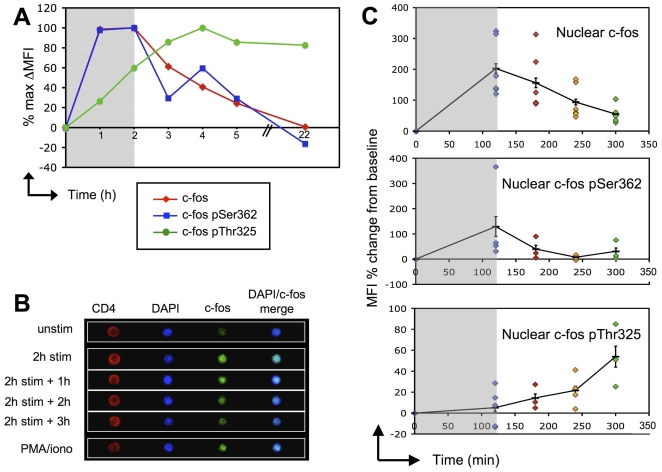
c-fos is rapidly induced and phosphorylated upon T cell stimulation and remains stable hours after stimulus withdrawal. Kinetics of c-fos total protein and its phosphorylated forms in CD4 T cells during a 2 h stimulation with anti-CD3/anti-CD28 beads and 1 h, 2 h, 3 h or 20 h after removal of beads as determined by intracellular staining and FACS analysis. In order to display data from all 3 stainings in a single graph, results are shown as the percent of the maximum change of fluorescence observed for each antibody, e.g. c-fos MFI of the sample/(max c-fos MFI observed in the time course-baseline c-fos MFI)×100. B. Representative images taken by an imaging flow cytometer of CD4 T cells stained intracellularly for c-fos total protein and DAPI. Cells were either left unstimulated or stimulated for 2 h with anti-CD3/anti-CD28 beads, then harvested immediately or 1 h, 2 h or 3 h after stimulus withdrawal. A positive control sample was stimulated with PMA/ionomycin for 2 h. C. LN cells were stimulated 2 h with anti-CD3/anti-CD28 beads and harvested either immediately or 1 h, 2 h or 3 h after removal of beads. The cells were stained for CD4 and for total c-fos protein (top panel) or its phosphorylated forms, pSer362 (middle panel) or pThr325 (bottom panel) before analysis on an imaging flow cytometer. Colocalization with DAPI was used to determine nuclear localization in CD4+ T cells. Each dot represents a different sample. Results (mean±sem) are shown as the percent change in mean fluorescence intensity (MFI) from baseline levels in unstimulated T cells and are pooled from at least 3 independent experiments.

Any mechanism underlying signal integration by T cells would imply that certain modifications induced by the first suboptimal TCR stimulation would persist after cessation of TCR signaling. While several non-mutually exclusive mechanisms could contribute to T cell biochemical memory, we hypothesized that upregulation of IEG products playing an important role during T cell activation, such as c-fos, may also fulfill these criteria. Thus, using both regular and imaging flow cytometry, we monitored intracellular c-fos levels in T cells before, during and after TCR stimulation. C-fos levels rapidly increased and reached a plateau after 1 h of stimulation with anti-CD3/anti-CD28 beads (Figure 3A). Importantly, c-fos staining remained elevated at least 3 h after removal of the beads. These kinetics were confirmed by imaging cytometry, which also demonstrated that the protein was predominantly intranuclear (Figure 3B and C). Therefore, the presence of increased c-fos several hours after TCR cessation could participate in signal integration by making subsequent TCR stimulation more efficient.

An interesting aspect of c-fos regulation is that both its induction and its stabilization can be promoted by activated ERK. The newly expressed protein is rapidly degraded unless it is stabilized by phosphorylation of Ser 362 and Ser 374 on the C-terminus, which can be performed by RTK and ERK, respectively [24], [25]. This phosphorylation exposes a DEF domain that promotes further phosphorylation events by ERK on Thr 325 and Thr 331 [26]. It was therefore possible that phosphorylated forms of c-fos could persist for a prolonged period of time upon cessation of TCR stimulation. Indeed, when T cells were stimulated for 2 h with anti-CD3/anti-CD28 beads and the levels of c-fos with phosphorylation at Ser362 (pSer362) or Thr325 (pThr325) monitored by intracellular staining, the kinetics of c-fos pSer362 induction and decay mirrored those of total c-fos protein, but those of c-fos pThr325 were delayed, further increasing after withdrawal of the stimulus and, importantly, remaining elevated even one day later (Figure 3A). Parallel experiments using imaging cytometry to measure intranuclear phospho-protein levels yielded similar results (Figure 3C).

In various cell types including T cells, c-fos has been implicated in the cell's ability to discriminate between transient and sustained ERK signaling [26], [27]. Transient ERK signaling will promote expression of c-fos but not stabilization of the protein, while sustained ERK signaling will promote expression of c-fos as well as its subsequent stabilization. Our results suggest an additional implication for this mode of regulation: Because the levels of c-fos, and in particular of a phosphorylated form, do not return to baseline for several hours after termination of TCR signaling, the stabilized c-fos may have a long enough half-life to remain in sufficient quantities to allow the cell to quickly resume its activation program upon reinitiation of signaling. Therefore, accumulation of IEG products, typified by c-fos, may contribute to the phenomenon of T cell short term biochemical memory.

### c-fos levels could act as a ‘counter’ during intermittent TCR stimulation in vivo

**Figure 4:**
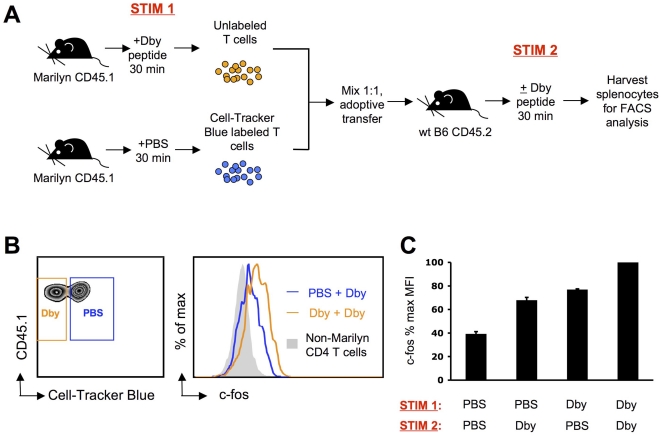
Induction of c-fos is additive during sequential in vivo antigen-specific stimulations. A. Experimental design: CD45.1 Marilyn TCR transgenic mice were injected with 5 µg Dby peptide and splenocytes and LN cells were harvested 30 minutes post-injection and left unlabeled. In parallel, control Marilyn mice were injected with PBS and splenocytes and LN cells were harvested 30 minutes post-injection and labeled with Cell-Tracker Blue. CD4+ T cells from both sources were purified by negative selection and mixed at a 1∶1 ratio before adoptive transfer into wildtype CD45.2 C57BL/6 congenic mice. Host mice received an injection of 5 µg Dby peptide or PBS, and splenocytes were harvested 30 minutes later for intracellular staining of c-fos followed by FACS analysis. B. Left panel: Upon adoptive transfer, Marilyn T cells can be identified in the host spleen based on CD45.1 expression. Furthermore, Marilyn T cells purified from mice that received peptide (orange box, +Dby) can be distinguished from those purified from mice that did not receive peptide (blue box, +PBS) based on Cell-Tracker Blue labeling (left panel). Right panel: A single stimulation with Dby induces upregulation of c-fos (blue line) but two sequential stimulations result in a further increase in c-fos levels (orange line). Shaded gray histograms show c-fos levels in endogenous CD4 T cells as a baseline comparison. C. Change in of c-fos protein levels in Marilyn T cells at the end of the second in vivo stimulation for the indicated combination of stimulation. Results are compiled from 2 independent experiments and shown as the percentage (mean±sem) of the maximum c-fos fluorescence observed in each experiments.

It remains to be determined whether c-fos levels can be additive upon sequential in vivo stimulations. An injection of 5 µg Dby peptide will effect a detectable upregulation of c-fos in the T cells of Marilyn TCR transgenic mice within 30 minutes, but c-fos protein levels are not yet maximal at this timepoint. We therefore injected Marilyn mice with Dby peptide, isolated T cells 30 minutes later, and co-transferred these, together with fluorescently labeled naïve Marilyn T cells, into wild-type hosts. The new hosts were then injected with Dby peptide for another 30 minutes before harvesting splenocytes for analysis of c-fos levels (Figure 4A). After the first in vivo peptide stimulation, c-fos levels increased in Marilyn T cells as expected (Figure 4B). Upon secondary peptide stimulation in the new host, these levels increased further, demonstrating summation of the two periods of stimulation. The naïve Marilyn T cells that were co-transferred also upregulated c-fos expression during the second in vivo peptide stimulation, but to a lesser extent than their prestimulated counterparts. In hosts that received PBS instead of a second dose of peptide, the elevated c-fos levels in prestimulated Marilyn T cells started to decay slowly, but to a level that was nevertheless above baseline. (Figure 4C). Thus, a short antigenic stimulation causes a moderate increase in c-fos levels that nevertheless decays slowly enough after termination of TCR signaling that restimulation can result in additive accumulation of this IEG product.

### General conclusions

The present study reports two important findings. First, expanding on previous in vitro studies [5], [6], we provide in vivo evidence that short periods of stimulation can imprint on a T cell a biochemical memory of that activation signal. Second, we have identified a putative role for the IEG product c-fos in providing T cells with such a property. We observed an early upregulation of c-fos in T cells that decayed slowly upon stimulus withdrawal both in vitro and in vivo and could be boosted by subsequent TCR stimulation. We propose that successive antigen recognition events may lead to the stepwise accumulation of c-fos, and possibly other IEG products, in T cells, providing them with a mechanism for signal summation. Future experiments manipulating the levels and the stability of IEG products including c-fos may help further elucidate how T cells collect and integrate their activation signals in vivo.

## Materials and Methods

### Ethics Statement

Mice were cared for in accordance with Pasteur Institute guidelines in compliance with European animal welfare regulations, and all animal studies were approved by the Pasteur Institute Safety Committee in accordance with French and European guidelines.

### Mice

Six-week-old C57BL/6 (B6) mice were purchased from Charles River Laboratories. Female Marilyn (anti-H-Y) TCR transgenic RAG-2^−/−^ CD45.1^+/+^ mice were obtained from the CDTA. Mice expressing the OT-II transgenic TCR were bred in our animal facility.

### Cell Preparation and Transfer

Lymph node cells were isolated by crushing the lymph nodes and passing the cell suspension through a 70 µm nylon cell strainer. Splenocytes were isolated by crushing the spleen and passing the cell suspension through a 70 µm nylon cell strainer (BD Falcon) followed by RBC lysis in ACK Lysis Buffer. Adoptive transfer was performed by i.v. injection.

### In vitro T cell stimulation

Splenocytes, lymph node cells, or in some cases purified T cells were plated in 24-well plates at 4×10^6^ cells/well or in 96-well round-bottom plates at 150,000 cells/well with anti-CD3/CD28 magnetic beads (Invitrogen) at a 1∶1 bead∶cell ratio, centrifuged briefly (1 minute) at 1200 rpm to collect the cells and beads at the bottom of the wells, and incubated at 37°C for the times indicated. Beads were removed by gently pipetting several times the contents of each well and passing them over a magnet (Invitrogen) twice. When a second stimulation was necessary, these cells were re-plated and new beads added.

### In vivo T cell stimulation

OT-II TCR transgenic mice were injected i.v. with 50 µg LPS, and 6 hours later, with 200 µg OVA^323–339^ peptide (ISQAVHAAHAEINEAGR) or PBS as a vehicle control. Marilyn TCR transgenic mice were injected i.v. with 5 µg Dby peptide (NAGFNSNRANSSRSS) or PBS as a vehicle control.

### CFSE and Cell-Tracker Blue Labeling

Cells were pelleted and resuspended in PBS at a concentration of 5×10^7^ cells/ml or less. An equal volume of dye diluted in PBS was added and mixed thoroughly. Final concentrations of dye were as follows: 5 µM CFSE for in vivo experiments, 1 µM CFSE for in vitro experiments, and 10 µM Cell-Tracker Blue. Cells were incubated with dye at 37°C for 10 minutes, then washed twice with RPMI/10% FCS.

### FACS Analysis

Cell suspensions were incubated at 4°C for 15 min with combinations of the following antibodies: anti-CD4, anti-CD8, anti-CD62L, anti-CD25, anti-CD44, anti-CD45.1, anti-CD45.2 (eBioscience). For intracellular staining, cells were fixed with freshly prepared Fixation/Permeabilization Buffer (eBioscience), washed twice with Permeabilization Buffer (eBioscience), stained with primary antibody at 4°C for 1 hour (anti-c-fos (Cell Signaling), anti-c-fos pSer362 (abcam), anti-c-fos pThr325 (BioSource)), washed twice with permeabilization buffer, stained with secondary antibody at 4°C for 1 hour (Dylight-488-conjugated AffiniPure F(ab')2 Fragment Donkey Anti-Rabbit IgG; Jackson ImmunoResearch), and washed twice again with Permeabilization Buffer.

### Imaging cytometry

Cells were stained as for FACS analysis and resuspended in PBS at a concentration of 5×10^7^ cells/ml. DAPI (Invitrogen) was used to visualize cellular nuclei. 8,000 cells were acquired for each sample and digital imaging was performed on a multispectral imaging flow cytometer (ImageStreamX, Amnis Corporation, Seattle, WA). The data were analyzed using the manufacturer's software (IDEAS, Amnis Corporation). Briefly, fluorometric compensation was digitally calculated based on single-stain controls. The single cells and cells in focus were selected based on a digital plot of Aspect Ratio with Area of brightfield images and of Gradient RMS of DAPI images, respectively. DAPI-positive cells were gated and total amount of c-fos intensity/cell was calculated, as well as the amount of nuclear c-fos intensity/cell.
